# Stock Index Prediction Based on Time Series Decomposition and Hybrid Model

**DOI:** 10.3390/e24020146

**Published:** 2022-01-19

**Authors:** Pin Lv, Qinjuan Wu, Jia Xu, Yating Shu

**Affiliations:** School of Computer, Electronics and Information, Guangxi University, Nanning 530004, China; lvpin@gxu.edu.cn (P.L.); 1913392058@st.gxu.edu.cn (Q.W.); 2013391057@st.gxu.edu.cn (Y.S.)

**Keywords:** stock index forecasting, CEEMDAN, ADF, ARMA, LSTM, hybrid model

## Abstract

The stock index is an important indicator to measure stock market fluctuation, with a guiding role for investors’ decision-making, thus being the object of much research. However, the stock market is affected by uncertainty and volatility, making accurate prediction a challenging task. We propose a new stock index forecasting model based on time series decomposition and a hybrid model. Complete Ensemble Empirical Mode Decomposition with Adaptive Noise (CEEMDAN) decomposes the stock index into a series of Intrinsic Mode Functions (IMFs) with different feature scales and trend term. The Augmented Dickey Fuller (ADF) method judges the stability of each IMFs and trend term. The Autoregressive Moving Average (ARMA) model is used on stationary time series, and a Long Short-Term Memory (LSTM) model extracts abstract features of unstable time series. The predicted results of each time sequence are reconstructed to obtain the final predicted value. Experiments are conducted on four stock index time series, and the results show that the prediction of the proposed model is closer to the real value than that of seven reference models, and has a good quantitative investment reference value.

## 1. Introduction

The stock index is calculated based on some representative listed stocks. To some extent, it can reflect price changes of the whole financial market, hence its use as an important indicator of the country’s future macroeconomic performance. Forecasting the stock index accurately is of paramount importance for reducing risks in decision-making, by providing some important reference information [1]. However, owing to the complexity of the internal structure and the variability of external factors, changes of the stock market are dynamic and uncertain, and forecasting the stock index has always been a challenge. Many stock forecasting models are mostly classified as either statistical or machine learning models [2]. Statistical models were first used to predict the stock market in finance, and have made some achievements. However, they assume a linear and stationary time series, which is inconsistent with the dynamic, non-linear characteristics of the real stock market, so they have great limitations. A deep learning model can overcome the defects of traditional statistical models in time series prediction but is easily affected by noise in some complex and dynamic financial systems, making it difficult to mine the hidden features of time series, resulting in poor learning ability and limited prediction accuracy.

Therefore, a single statistical or machine learning model cannot well predict the stock index. To overcome these limitations, we propose a hybrid stock index forecasting model based on Complete Ensemble Empirical Mode Decomposition with Adaptive Noise (CEEMDAN) [3]. In this model, CEEMDAN is first used to decompose the original financial time series into a series of Intrinsic Mode Functions (IMFs) and a residual term. Then, the stability of the IMFs and the residual term is characterized using the Augmented Dickey Fuller (ADF) method, the low-volatility time series are classified as linear components, and high-volatility time series are classified as non-linear components. In the final step, the Autoregressive Moving Average (ARMA) model is applied to the linear component, and Long Short-Term Memory (LSTM) is applied to the non-linear component. The final prediction result is obtained by reconstructing each prediction series. This method makes full use of ARMA in linear problems and uses LSTM to identify and abstract non-linear features, mining the movement rules of hidden components in time series and improving prediction accuracy. Hence, our proposed method is referred to as CAL (CEEMDAN-ARMA-LSTM). In the CAL model, CEEMDAN sequence decomposition can reduce the complexity of time series, and the sequences that pass the ADF stationarity test have significant linear trends. Therefore, we employ ARMA to predict the data of the linear part, avoiding the waste of effective information caused by differential operation.

The hybrid model combining linear and non-linear methods has great advantages in time series prediction [4]. Ref. [5] proposed a hybrid time-series prediction model taking the residual generated by Autoregressive Integrated Moving Average (ARIMA), combining the differences in a non-stationary time series with ARMA, as the input of LSTM for fitting. The ARIMA-LSTM model has achieved more accurate forecasting results than the individual LSTM and ARIMA models. A moving average filter was used to decompose a time series into linear and non-linear components [6]. ARIMA and Artificial Neural Network (ANN) were used to model low- and high-volatility data, respectively. This hybrid ARIMA-ANN model can achieve good prediction results. Each hybrid model in the literature combined linear and non-linear models in different ways, providing different perspectives for time series data prediction. However, these methods have the limitations that the error sequence generated by a linear model is assumed to be non-linear [5], and the original sequence is decomposed into single linear and non-linear components, which cannot mine the internal features of an overly complicated time series [6].

Our proposed model can properly decompose the original time series, and the ARMA and LSTM models are applied, which overcomes the defects of strong assumptions [5] and insufficient decomposition [6]. We validate our model’s effectiveness on four stock market indices. The experimental results show that the proposed model has higher prediction accuracy than seven reference models on these indices. The main contributions of this study are summarized as follows:

1. The advantages of CEEMDAN are used to decompose the original complex sequential data into trends of different scales. This reduces the complexity of the original time series to extract abstract and deep features.
2. The ADF test method effectively combines the linear and non-linear models. This method can judge the stationarity of data. The linear prediction method of ARMA is used for the stationary time series, and the non-linear prediction method of LSTM for unstable time series.
3. The proposed CAL model is compared with the individual LSTM, Gated Recurrent Units (GRU), Bi-directional LSTM (Bi-LSTM), ARIMA models and the hybrid EMD-ARMA-LSTM, CEEMDAN-LSTM [7], and ARIMA-ANN [6] models. Experiments on different datasets show that the CAL model outperforms traditional hybrid models, improved deep learning model, and their separate component models.

The remainder of this article is organized as follows. Section 2 summarizes related work. Section 3 introduces the proposed CAL model. Section 4 experimentally evaluates the proposed method on real stock index datasets. Section 5 summarizes the paper and points out future research directions.

## 2. Related Work

Time series analysis is an important tool in many stock market prediction methods, and it makes predictions by analyzing observed points in the series. As one of the most widely used linear time series forecasting methods, the ARIMA model [8] integrates the Autoregressive (AR) and Moving Average (MA) models. It assumes that future predictions have a linear dependence on the current and past data values. Therefore, ARIMA can only fit linear stationary time series data; the non-stationary time series might not be modeled effectively.

Deep learning can overcome the limitations of traditional linear models, such as weak fitting ability and weak feature extraction ability with non-linear data, and has gradually become a key research method in stock prediction. Some deep learning models, such as Convolutional Neural Networks (CNNs), can identify non-linear relationships and extract hidden information from data. LSTM can retain long historical information and achieve high prediction accuracy in sequential pattern learning problems. It does not require selecting features manually [9] and the performance to be superior to that of Feedforward Neural Network (FNN) [10], a Deep Neural Networks (DNN) [11], and Support Vector Machines (SVM) [12]. Although deep learning well models some complex problems, the traditional linear model still has some advantages. For example, the regression method sometimes has better prediction performance than deep learning in power system prediction [13,14].

Based on the above analysis, no individual model can be applied well in all circumstances. In a practical problem, the appropriate model depends on the characteristics of the dataset. However, in time series prediction, it is sometimes difficult to define whether the data are linear or non-linear, especially when there are multiple linear or non-linear components, making it difficult to choose an appropriate prediction model.

Various hybrid techniques exploit the unique strengths of both types of model to effectively improve prediction performance [4,5,6]. Ref. [15] combined ARIMA and SVM, which showed that the combined model was better than either of its components at stock price prediction. LSTM and an Autoregressive Conditional Heteroscedasticity (GARCH) model were combined to predict stock price volatility, with relatively accurate results [16]. Ref. [17] proposed an ARIMA-ANN hybrid model to improve time series predictions when a time series has both linear and non-linear components. Ref. [18] developed three different hybrid models combining linear ARIMA and non-linear models, such as SVM, ANN, and random forest (RF) models, to predict stock index returns. Experimental results showed that the hybrid model ARIMA-SVM achieved the highest accuracy and the best return.

## 3. Stock Index Forecasting Model

### 3.1. Related Models

#### 3.1.1. CEEMDAN

Empirical mode decomposition (EMD) [19] can decompose time series data into subseries according to their own time scales without setting a basis function, for effective treatment of non-linear and unstable data. However, mode aliasing can occur during EMD data decomposition. Ensemble Empirical Mode Decomposition (EEMD) addresses this problem but cannot completely eliminate reconstruction error after the introduction of Gaussian white noise [7]. In the process of decomposition, CEEMDAN adaptively adds white noise to avoid mode mixing of EMD, and addresses reconstruction error due to noise. The prediction of stock prices is affected by multiple factors and is a non-linear complex model. The components of CEEMDAN are relatively simple; hence, more accurate predictions can be obtained.

#### 3.1.2. LSTM

As a special recurrent neural network, LSTM solves the problem of gradient disappearance and explosion in the training process of long sequences, and it has a more complex network structure. LSTM introduces a cellular state and combines forgetting, input, and output gates to discard, maintain, and update information. The output of the model is calculated by multiple functions involving some summation operations, so it is not easy to produce the problems of gradient disappearance and explosion in the process of backpropagation. LSTM has advantages in some problems related to time series, such as industrial time series prediction [20] and text translation [21]. We take this model as the non-linear part of time series prediction.

#### 3.1.3. ARMA

ARMA is a linear sequential method that predicts a future according to historical and current data. ARMA data prediction must meet the requirements of stationarity. In practice, trends and periodicity often exist in many datasets, so there is a need to remove these effects before applying such models. Removal is typically carried out by including an initial differencing stage in the model, and the model is transformed into an ARIMA model. Therefore, ARIAM can be seen as an enhanced version of ARMA. It has a wider range of applications but a certain amount of information loss.

### 3.2. Proposed Model

It is widely accepted that the financial market is complex and dynamic, which calls for a noise elimination or time series decomposition. For this purpose, a multi-scale decomposition method called CEEMDAN is used in our model. The decomposed components have different scales; ARMA and LSTM are used as linear and non-linear prediction modules to exploit their respective advantages. Thus, a hybrid ARMA-LSTM model for time series forecasting based on CEEMDAN is proposed, which is called CAL (CEEMDAN-ARMA-LSTM). CEEMDAN can adaptively decompose a time series, yielding a series of IMFs and residue with different characteristic scales. The decomposition principle is given by

(1) $$s\left(t\right)=\sum_{i=1}^{n}imf_{i}\left(t\right)+res\left(t\right),$$

where s(t) represents given time series data; $imf_{i}\left(t\right)$ (*i* = 1, 2,…, *n*) represents the different IMFs; and *res*(*t*) is the residue. Each IMF and residue has its own local characteristic time scale. A low-volatility sequence contains more linear features, and ARMA is more suitable for processing. A high-volatility sequence can be considered non-linear, which better suits LSTM. We require a method to separate the linear and non-linear components and feed them into ARMA and LSTM.

Each hybrid model brings its own perspectives to time series decomposition. We use a statistical ADF method to separate linear and non-linear components. The ADF test can identify whether a time series is stationary. The existence of a unit root in a sequence indicates that a series is unstable. A more negative ADF test result indicates more stable data, and 0.05 is an accepted threshold to judge the stability of a dataset, which can used to separate linear and non-linear sequences [4].

(2) $$s\left(t\right)=\sum_{i=1}^{m}l_{i}+\sum_{i=m+1}^{n+1}n_{i}.$$

An ADF stationary test separates time series decomposed by CEEMDAN in Equation (2), where $l_{i}$ and $n_{i}$, respectively, denote linear and non-linear components.

(3) $$L_{t}=g\left(l_{t-1},l_{t-2},\ldots ,l_{t-p},\varepsilon_{t-1},\varepsilon_{t-2},\ldots ,\varepsilon_{t-q}\right).$$

After the linear and non-linear components, respectively. The modeling process of ARMA is described by Equation (3), where $l_{t-1}$ to $l_{t-p}$ are time sequence values of the past p days, $\varepsilon_{t-1}$ to $\varepsilon_{t-q}$ denote corresponding random error, and *g* is the linear function of ARMA. It can be seen from Equation (3) that the results are related to the sequential values and random errors in a past period of time, so it can be concluded that its prediction process can reflect the continuity of the original sequence in time.

LSTM can mine the characteristics of non-linear time series, which we use to fit non-stationary sequences.The LSTM modeling process is described by Equation (4), where *f* is the non-linear function of LSTM, and *a* is the number of days observed by the model, i.e., how far we will go back in time. The prediction results of the linear and non-linear parts are obtained by the corresponding models, and the final prediction is the integration of the linear and non-linear parts in Equation (5), where y(t) denotes the final predictions.

(4) $$N_{t}=f\left(n_{t-1},n_{t-2},\ldots ,n_{t-a}\right),$$

(5) $$y_{t}=\sum_{i=1}^{m}L{}_{i}+\sum_{i=m+1}^{n+1}N_{i}.$$

**Figure 1:**
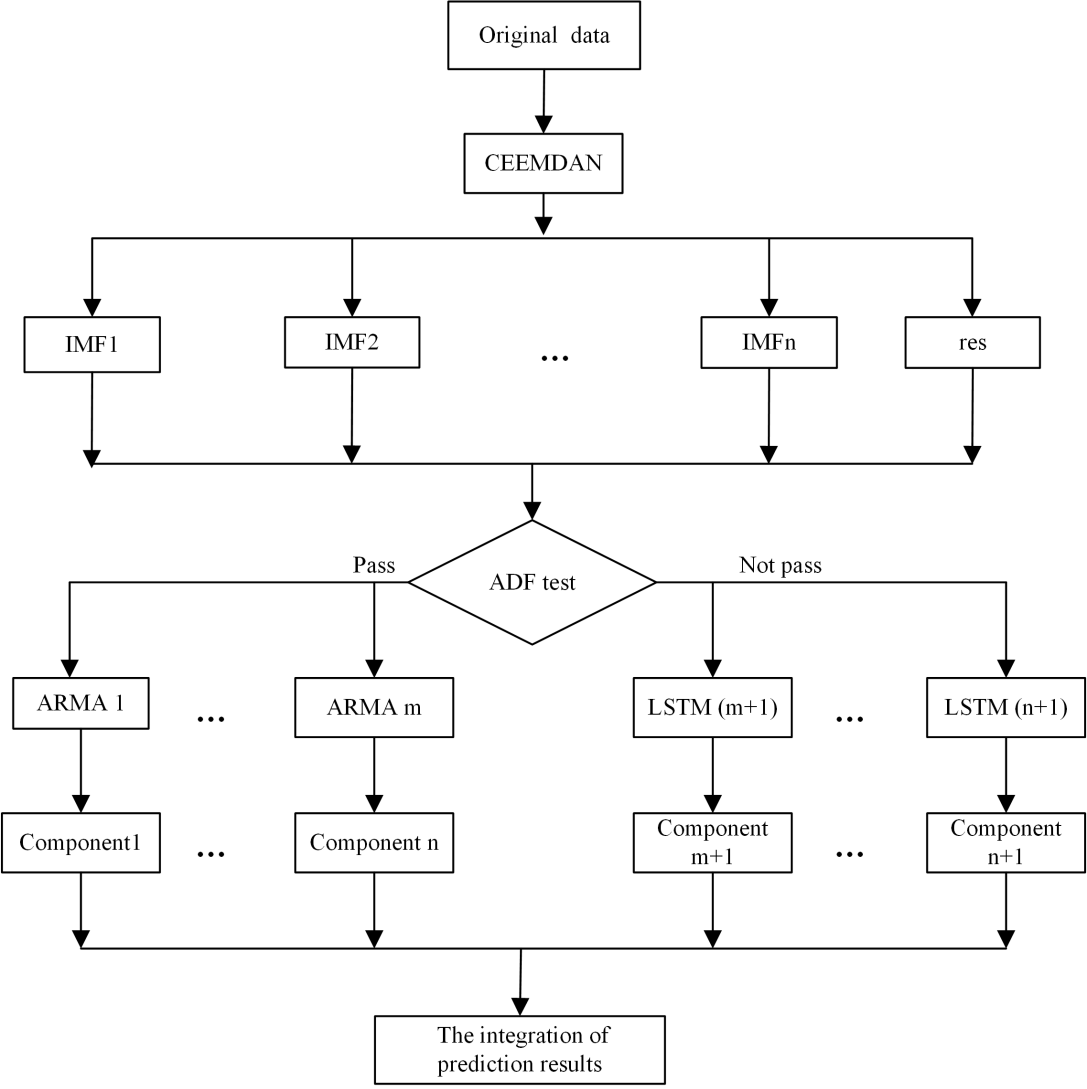
Stock market index forecasting model.

To sum up, the CAL model prediction consists of time series decomposition, an ADF stationary test, model fitting, and integration of results. Figure 1 shows the prediction model, where IMF${}_{1}$-IMF${}_{n}$ are IMF components after time series decomposition, and res is the residue. ARMA${}_{1}$-ARMA${}_{m}$ denote that the *m* sequences pass the ADF test and are fitted using ARMA, and LSTM${}_{\left(m+1\right)}$-LSTM${}_{\left(n+1\right)}$ denote the n−m+1 sequences that fail the ADF test and are modeled by LSTM. The steps of the proposed hybrid model are as follows.

1. Given time series decomposition, using a CEEMDAN method (Equation (1)), time series data are decomposed into finite IMFs and residue. Components can be more or less volatile.
2. Sequences with different stability are separated by an ADF stationary test (Equation (2)).
3. Low- and high-volatility components are fitted by ARMA (Equation (3)) and LSTM (Equation (4)), respectively.
4. The final result is the sum of the predictions of each component (Equation (5)).

## 4. Experimental Results and Discussions

In this section, we experimentally present the predictive ability of the CAL model. In Section 4.1, datasets used in experiments are introduced. In Section 4.2 and Section 4.3, the evaluation metrics and parameter settings in the experiment are discussed, respectively. The decomposition results of EMD and CEEMDAN are compared in Section 4.4. The models for comparison are listed in Section 4.5. The predicted effects of the CAL model and other comparative methods are evaluated in Section 4.6.

### 4.1. Datasets

We use one-step-ahead prediction to verify the prediction accuracy of the proposed CAL model on four major global stock indices: Deutscher Aktien (DAX), Hang Seng (HSI), Standard and Poor’s 500 (S&P500), and Shanghai Stock Exchange Composite (SSE). These have strong representation in the global financial market and can reflect stock market changes, which has much research value. Stock market indices are affected by national policies, market environments, and other factors presenting different characteristics. Research on stock market indices in different financial markets can examine the prediction accuracy of the model.

The dataset comes from Yahoo! Finance. The range of each stock index is from 13 December 2007, to 12 December 2020, and the daily closing price is selected as the research object. The first 90% of the dataset in the time order of each stock index is used as the training set, and the last 10% is used as the test set. Only the data of trading days are used for research.

**Figure 2:**
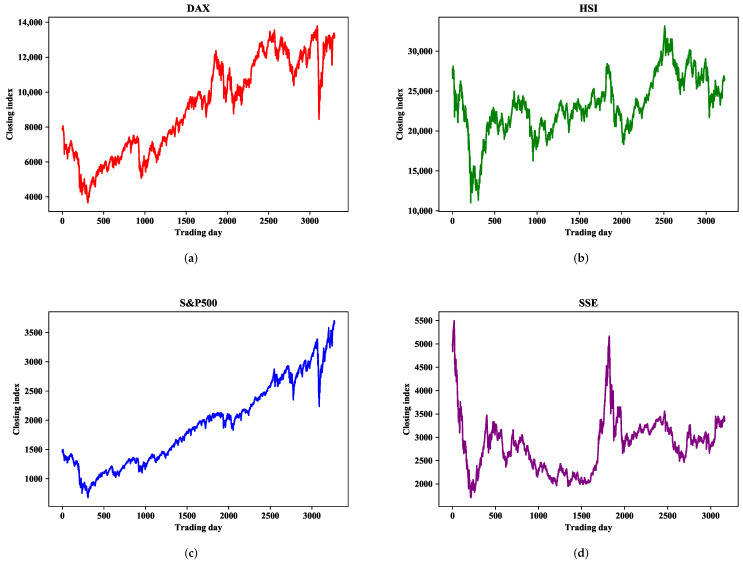
Daily closing index series of four financial markets. (**a**) DAX. (**b**) HSI. (**c**) S&P500. (**d**) SSE.

The statistical analysis of each stock index is shown in Table 1, where we determine the amount of data contained in each stock market index, as well as the average, maximum, minimum, standard deviation, and ADF test results of the closing index. As can be seen from Table 1, there is a large gap between the maximum and minimum values, and a large standard deviation, indicating that these closing indices have great volatility within the research range. Moreover, the ADF test results of the DAX and S&P500 are greater than the threshold 0.05, indicating that the dataset is highly volatile and non-stationary. SSE is somewhat more stable than the other three datasets. Figure 2 shows the sequential change of the closing index within the study range, from which it can be seen that the four indices all have great volatility and instability in the short term.

### 4.2. Evaluation Metrics

We evaluate the proposed CAL model by the Mean Absolute Error (*MAE*), Root Mean Square Error (*RMSE*), Mean Absolute Percentage Error (*MAPE*), and R-squared (*R*${}^{2}$), defined as Equation (6) to Equation (9).

(6) $$MAE=\frac{1}{n}\sum_{i=1}^{n}|p_{t}-y_{t}|$$

(7) $$RMSE=\sqrt{\frac{1}{n}\sum_{t=1}^{n}(p_{t}-y_{t}{)}^{2}},$$

(8) $$MAPE=\frac{1}{n}\sum_{t=1}^{n}|\frac{p_{t}-y_{t}}{y_{t}}|\times 100,$$

(9) $$R^{2}=1-\frac{\sum_{i=1}^{n}{\left(p_{t}-y_{t}\right)}^{2}}{\sum_{i=1}^{n}{\left(p_{t}-\bar{y_{t}}\right)}^{2}}.$$

Here, $p_{t}$, $y_{t}$, and $\bar{y_{t}}$ are the predicted, actual, and average of actual values, respectively, and *n* is the prediction horizon. *MAE* measures the average magnitude of the errors in a set of predictions, without considering their direction. *RMSE* is a quadratic scoring rule that also measures the average magnitude of the error. It is the square root of the average of squared differences between prediction and actual observation. MAPE measures the percentage error of the forecast in relation to the actual values. *R*${}^{2}$ is a statistical measure in a regression model that determines the proportion of variance in the dependent variable that can be explained by the independent variable. It corresponds to the squared correlation between the observed values and the predicted values by the model. A higher value of *R*${}^{2}$ means a better prediction accuracy.

### 4.3. Parameter Settings

**Figure 3:**
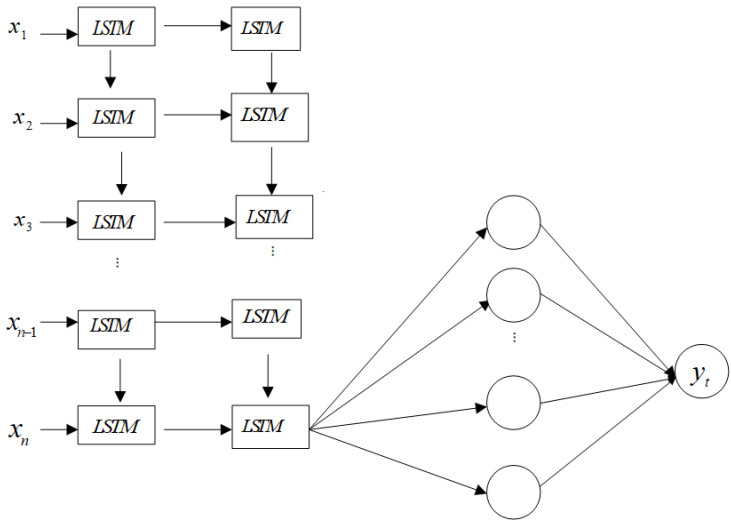
LSTM network architecture.

The sequential model structure in Keras is used to build the LSTM network. The batch size of the model is 128. Two layers of LSTM are employed to build the sequential model, and the output of the second layer of the last LSTM unit is connected to a fully connected layer. Then, the fully connected layer is connected to another fully connected layer for the final output. Figure 3 shows the LSTM network structure, where $x_{i}$ (*i* = 1, 2,…, *n*) is the input to the model. The numbers of units in each LSTM in the first and second layers are 128, 64, respectively. The third fully connected layer has 16 neurons, and the last layer has only one unit, which will provide a predicted value. Fully connected units and LSTM units use the ReLU and tanh activation function, respectively. We use MSE as a loss function, and use Adam as an optimization algorithm. Adam is an adaptive learning rate optimization algorithm that utilizes both momentum and scaling, and it has two decay parameters that control the decay rates and adjust the learning rate adaptively [22]. We explore the influence of different training epochs on the experimental results, and the results suggest that more training epochs result in a more skillful model, but it may lead to the problem of overfitting. Therefore, it is suitable to set the epoch to 200. The time steps works best at 10. The detailed parameter settings are shown in Table 2.

The best fitted ANN of ARIMA-ANN model in comparison has a layered architecture of 17 × 17 × 1 [4]. The parameters of CEEMDAN-LSTM refer to Ref. [7]. The parameters of LSTM, GRU, and Bi-LSTM, in comparison, are similar to that of LSTM in the CAL model.

Grid search is used to determine the optimal parameters *p* and *q* of the ARMA model. The range of the grid search is [0, 5], and the group with the smallest Akaike Information Criterion (AIC) value is selected.

### 4.4. Decomposition Results of EMD and CEEMDAN

Stock indices, which contain many influencing factors, can be decomposed used EMD or CEEMDAN. We take the SSE stock index as an example to decompose the original time series, so as to compare the two decomposition methods. To intuitively compare the results, we limit CEEMDAN and EMD to generate the same number of IMFs.

**Figure 4:**
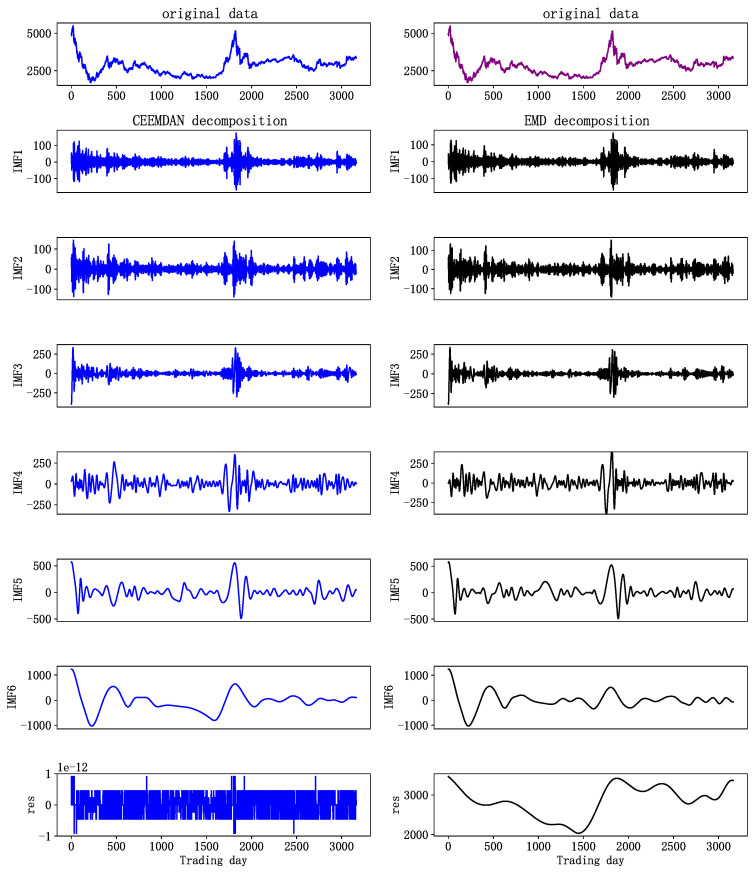
SSE decomposition results.

In Figure 4, the decomposing results of the original SSE index series are demonstrated. The results of sequence decomposition range from high to low frequency. The first few IMFs, with more noise, represent the high-frequency components in the original data; the middle IMFs, with reduced frequency, represent middle-frequency components; and the last few IMFs, with less volatility, which is similar to the long-term movement trend of a stock, represent the low-frequency components. The left and right sides of Figure 4 show the results of CEEMDAN and EMD data decomposition, respectively. It can be found that IMF5 and IMF6 on the right of Figure 4 have similar scales and are not easily distinguished. This is because the mode aliasing of EMD leads to the distribution of some similar time scales in different intrinsic mode functions, resulting in waveform aliasing and mutual influence. As a result, the features of a single sequence are not obvious, and feature extraction of later prediction models is more difficult. CEEMDAN data decomposition effectively solves this problem. As can be seen from the decomposition results on the left side of Figure 4, CEEMDAN decomposed the stock index into several components, from high- to low-frequency, whose characteristics are obvious, and there is no waveform aliasing.

### 4.5. Comparative Models

To verify the effectiveness of the proposed CAL model for stock market prediction, we experimentally compare seven models. Table 3 lists the models and reference purposes of these seven controlled experiments, which verify the proposed model from different perspectives.

1. LSTM deep learning model: LSTM networks can automatically detect the best patterns suitable for raw data, and are widely utilized in financial time series modeling [23,24,25]. However, LSTM methods are susceptible to noise. The comparison result of CAL and LSTM can evaluate whether the proposed model can effectively improve the results of LSTM in complex time series modeling.
2. Linear ARIMA model: ARIMA can better predict linear time series, but is not suitable for complex non-linear time series [4]. We combine ARMA and LSTM to extend the application range of the ARIMA time series model. In addition, the prediction effects of the ARIMA and CAL models are compared, which verifies the effectiveness of the proposed model compared with a single linear model.
3. GRU: GRU is a simplified version of the LSTM. It uses only one state vector and two gate vectors, i.e., reset gate and update gate. The comparison result can evaluate whether the CAL model is better than other deep learning model.
4. Bi-LSTM: To preserve the future and the past information, Bi-LSTM makes the neural network have the sequence information in both directions, i.e., backwards (future to past) and forward (past to future). The aim of the experiments is to show whether Bi-LSTM improves the prediction accuracy of LSTM. The experiments also verify the effectiveness of the proposed model compared with a single improved model.
5. EMD-ARMA-LSTM model: EMD can generate more predictable components when fed into the decomposing module. CEEMDAN is designed to solve the problem of EMD mode mixing. To compare the prediction effects of EMD-ARMA-LSTM, and CAL, we verify the influence of different decomposition methods on model prediction.
6. Hybrid ARIMA-ANN model [6]: ARIMA and ANN are adopted to model the linear and non-linear data [6], and empirical results demonstrate that ensemble models can effectively improve performance. We use the ARIMA-ANN model for comparison. The results can demonstrate the advantages of CAL over ARIMA-ANN when combining linear and non-linear models. The advantages of LSTM over an ANN in abstract feature extraction and prediction ability could also be verified.
7. CEEMDAN-LSTM model [7]: The CEEMDAN-LSTM model integrates the advantages of CEEMDAN and LSTM but does not consider that the original time series may contain linearly correlated components, and the non-linear prediction of all decomposed sequences will affect the prediction performance of the model. The empirical results demonstrate the validity of the CAL model in comparison to the CEEMDAN-LSTM model.

### 4.6. Experiments and Discussions

**Figure 5:**
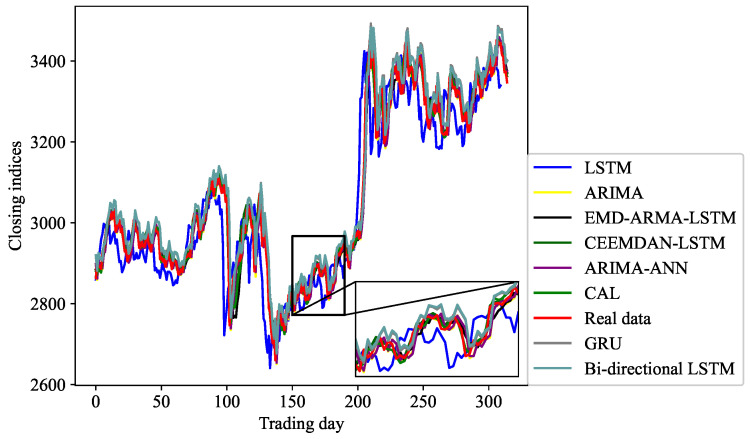
SSE comparison of sequence prediction results.

**Figure 6:**
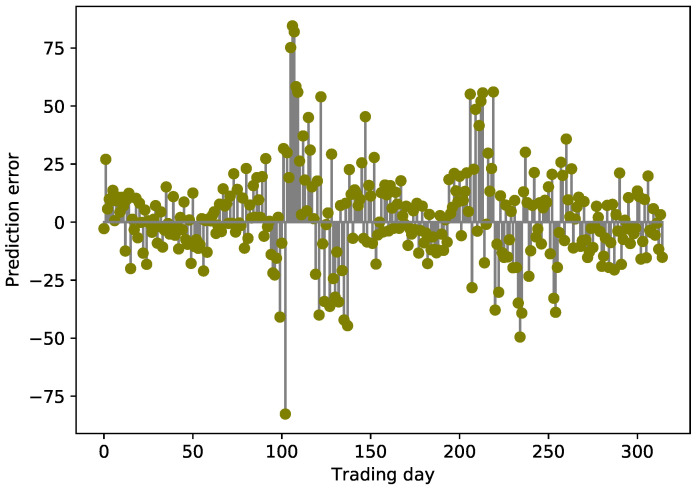
SSE error changes between real and predicted values.

We verify the effectiveness and superiority of the proposed model from three aspects:

1. Statistics of MAE, RMSE, MAPE, and R${}^{2}$ are chosen to assess the consistency between predicted and observed terms. These indicators measure the deviation between forecast and reality from different aspects.
2. The deviation between real and predicted values can be observed from Figure 5, and the variation of the error can be utilized to observe the stability of the CAL model from Figure 6.
3. A linear regression model is then used to further observe the performance of the CAL model; then, a series of technical diagnostics are leveraged to check the regression models.

#### 4.6.1. Observation of the Statistical Data

It can be observed from Table 4 that the CAL model has obvious advantages in stock index DAX series prediction, which decreases by 56.71% when compared to LSTM, and by 46.83% when compared to ARIMA in MAE. This indicates that a single model cannot effectively capture data patterns and make excellent predictions. Although GRU and Bi-LSTM improve the prediction accuracy of LSTM, their prediction accuracies are still lower than CAL.

Methods with EMD achieve remarkably less error in their forecasts than CEEMDAN-LSTM and CAL, which shows that experimental results vary with data decomposition, and CEEMDAN-based methods can achieve better predictive performance. The ARIMA-ANN model is inferior to EMD- and CEEMDAN-based methods, perhaps because it has limited decomposition ability to extract hidden features. CEEMDAN properly decomposes time series, reduces their complexity, and improves LSTM information extraction, so the hybrid CEEMDAN-LSTM model can achieve a better prediction effect than just LSTM. However, CEEMDAN-LSTM is not as good as CAL because it does not consider linear factors that may exist in the original sequence in time series prediction.

Table 5 lists the prediction performance of different models on the HSI stock index, where we find a large error between the real and predicted values. This is mainly because the data of the HSI stock index are more volatile and difficult to predict. The CAL model achieves the best prediction accuracy, followed by CEEMDAN-LSTM, EMD-ARMA-LSTM, and ARIMA-ANN. ARIMA-ANN achieve higher prediction accuracy than the individual ARIMA and LSTM models, and ARIMA obtains better results than LSTM. As deep learning is easily affected by noise, it is difficult to learn effective data patterns in complex dynamic time series. Deep learning methods, such as LSTM, GRU, and Bi-LSTM, have the largest prediction error on the HSI stock index. Although ARIAM has a higher prediction accuracy than them, the gap between predicted and actual values of ARIAM is still large. This indicates the predictive performance of a single model is very limited. The hybrid model performs better than the single ARIMA and LSTM models. The experimental results show that ARIAM-ANN gives poorer results than CEEMDAN-LSTM, EMD-ARMA-LSTM, and CAL, perhaps due to an insufficient scale of decomposition. CEEMDAN-LSTM and EMD-ARMA-LSTM effectively improve prediction accuracy, but the effect is still inferior to the proposed CAL model, which has advantages and good potential in high-volatility time series data.

Figure 2c shows that the movement trend of S&P500 is relatively stable, with little fluctuation in the research interval, and an overall upward trend. Hence, the predicted results are closer to the observed values of stock indices. Table 6 shows the experimental results of S&P500. The data show that the CAL model yields the smallest prediction error, with MAE 48.84% less than LSTM and 49.75% less than ARIMA. This shows that the single model has better prediction performance in some stable time series sets, but there is still room for improvement. However, GRU and Bi-LSTM cannot effectively improve the prediction accuracy. The prediction effect of EMD-ARMA-LSTM is still inferior to that of CAL, which further demonstrates the superiority of CEEMDAN over EMD data decomposition. CEEMDAN-LSTM achieves better prediction performance than the single LSTM model, and ARIMA-ANN yields higher prediction accuracy than ARIAM, showing that sequence decomposition and model combination can improve the prediction accuracy of financial series.

Table 7 shows the prediction performance results for SSE datasets. From Table 7, we can see that CAL has better predictive accuracy than the other seven models, with MAE up to 14.0294, followed by CEEMDAN-LSTM and EMD-ARMA-LSTM. ARIMA can achieve higher prediction accuracy than ARIMA-ANN and EMD-ARMA-LSTM. GRU and Bi-LSTM achieve higher prediction accuracy than LSTM.

Several important results are obtained on the SSE dataset. GRU and Bi-LSTM outperforms LSTM, but their prediction results are lower than ARIMA, which shows that a linear model can sometimes achieve a better prediction effect than a deep learning model. The prediction accuracy of EMD-ARMA-LSTM is relatively low, perhaps because the mode mixing of EMD leads to the inclusion of other scales of data in an IMF, and these abnormal data interfere with information extraction.

#### 4.6.2. Prediction Results and Errors

As demonstrated in Figure 5, we zoom in a part of the prediction interval to observe the consistency between the real and predicted values of different models. It can be seen that the CAL model yields the closest prediction results, and CEEMDAN-LSTM is closer to the observed values in comparison with EMD-ARMA-LSTM and ARIAM. LSTM and GRU have larger volatility and prediction error than the other models. The stem diagram oscillates up and down around the zero axis in Figure 6 and is locally symmetrical concerning the zero axis, indicating that the prediction results of the CAL model are relatively stable within the prediction interval.

#### 4.6.3. Regression Analysis

We conduct a linear regression to assess the correlation between the real data and the predicted values. The predicted value is denoted as *x*, and the real value is *y*, respectively. The regression equation is y=ax+b. The metrics, including standard error (SE), *p*-value (*p*) and *t*-value (*t*), are used to test the results of regression analysis. The definitions of SE and *t* are as follows, and *p* is derived from the *t* distribution.

(10) $$SE=\frac{\sigma }{\sqrt{n}},$$

(11) $$t=\frac{\bar{x}-\mu }{\frac{\sigma }{\sqrt{n}}}.$$

**Figure 7:**
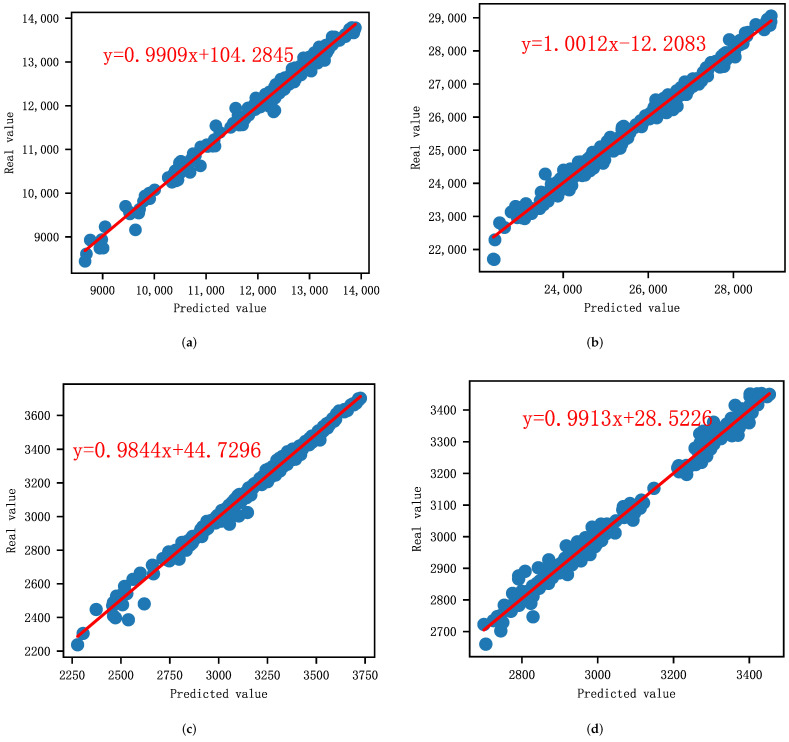
Linear regression analysis. (**a**) DAX. (**b**) HSI. (**c**) S&P500. (**d**) SSE.

Here, σ is the standard deviation of the predicted values, *n* is the number of the predicted (or real) values, $\bar{x}$ is the mean of the predicted values, and μ is the mean of real values. Table 8 lists the regression parameters and diagnostics results. It is observed that the slope *a* of each stock index is close to 1, the SE for *a* is relatively small, which means that the predicted values are very close to the real values. Furthermore, for each linear regression model, *p* for *a* is below the standard cutoff of 0.05, and *t* for *a* is high, suggesting it is a good model. In addition, Figure 7 shows the linear regression results of each stock index. The scattered points are evenly distributed near the fitting line, which indicates that the predicted and real values are highly correlated.

#### 4.6.4. Summary

Based on the above experiment results, the observations are summarized as follows.

1. Our proposed CAL model, with CEEMDAN-based methods, outperforms seven benchmark models in predictive accuracy on four stock indices from different developed stock markets, which indicates that methods with multi-scale decomposition can reduce the complexity of sequences, extract hidden features, and improve prediction accuracy.
2. CAL can obtain predictions closer to real values than CEEMDAN-LSTM, which indicates that components after decomposition may have both linear and non-linear characteristics. Therefore, models combining ARMA and LSTM can obtain more accurate predictions than individual LSTM models.
3. CAL can yield the closest prediction results in comparison to ARIMA-ANN. This indicates that the CAL model has advantages over some traditional hybrid models.
4. The prediction results show that CAL has a smaller prediction error than EMD-ARMA-LSTM does, and this indicates that the CEEMDAN method is superior to EMD in data decomposition.
5. In some volatile financial markets, a single prediction model, even improved deep learning model, has limited prediction ability because they cannot excavate internal movement rules of time series and reflect the multi-scale characteristics of financial time series.
6. The linear regression analysis shows the strong correlation between the predicted values and the real values, and the proposed prediction model is effective.

## 5. Conclusions and Discussion

Stock market index prediction plays an important role in reflecting overall stock market trends and has strong practical investment value. We proposed a hybrid stock index prediction model based on CEEMDAN and ARMA-LSTM. It takes the strengths of CEEMDAN in data decomposition, combines linear and non-linear models, and can well model complex time series. To verify the effectiveness of the prediction model, CAL was used to forecast the closing index of four stock markets, and seven control experiments were conducted for comparison. The results show that CAL can achieve the highest prediction accuracy. To optimize the model, future research can be conducted from the following aspects.

1. Single data source analysis has certain limitations. Combined analysis with different data sources, such as text information [26], can improve prediction to a certain extent.
2. Stock market data contain noise that affects forecast results. Methods, such as wavelet denoising [27] and principal component analysis [28], can eliminate the influence of irrelevant factors and improve the prediction effect to a certain extent.
3. Time series analysis has been applied in fields, such as natural science [29] and industrial time series prediction [30]. The application scope of the temporal sequence model in this paper can be extended, especially in some complicated temporal sequence scenes.

## Figures and Tables

**Table 1:** Descriptive statistics of closing indices.

Index	Count	Mean	Max	Min	Standard Deviation	ADF Test
DAX	3300	9118.21	13,789.00	3666.41	2722.52	0.79
HSI	3219	23,206.70	33,154.12	11,015.84	3660.60	0.11
S&P500	3273	1915.40	3702.25	676.53	713.03	0.99
SSE	3163	2846.43	5497.90	1706.70	586.51	0.01

**Table 2:** Details of the parameters of the CAL model.

Parameter	Meaning	Value
Input layer	Number of input layer nodes	128
Hidden layer 1	Number of first hidden layer nodes	64
Hidden layer 2	Number of second hidden layer nodes	16
Output layer	Number of output layer nodes	1
Batch size	Pass through to the network at one time	128
Optimization algorithm	Select the training mode	Adam
Loss function	With the goal of minimizing the loss	MSE
Epochs	Number of training	200
Timesteps	Input time steps	10

**Table 3:** Contrastive experiments.

Model	Comparison Purpose of Model Settings
LSTM	Comparison to single deep learning model
ARIMA	Comparison to single linear model
GRU	Comparison to other single non-linear model
Bi-LSTM	Comparison to improved deep learning model
EMD-ARMA-LSTM	Evaluation of CEEMDAN and EMD
ARIAM-ANN	Comparison of CAL to hybrid models [6]
CEEMDAN-LSTM	Comparison of CAL to stock forecasting model [7]

**Table 4:** Prediction results of different models in DAX.

Model	MAE	RMSE	MAPE (%)	R${}^{2}$
LSTM	167.0816	224.5003	1.4006	0.9570
ARIMA	136.0422	206.5253	1.1633	0.9650
GRU	153.5215	216.7465	1.2982	0.9608
Bi-LSTM LSTM	138.0041	209.2315	1.1768	0.9641
ARIMA-ANN	140.4099	211.9800	1.1966	0.9630
CEEMDAN-LSTM	97.2277	128.2331	0.8106	0.9866
EMD-ARMA-LSTM	127.1255	191.0622	1.0771	0.9687
CAL	72.3340	101.8321	0.6099	0.9915

**Table 5:** Prediction results of different models in HSI.

Model	MAE	RMSE	MAPE (%)	R${}^{2}$
LSTM	257.7703	347.1944	1.0197	0.9454
ARIMA	250.9188	345.3399	0.995	0.9470
GRU	256.1635	345.9382	1.0134	0.9451
Bi-LSTM	258.2292	353.4523	1.0249	0.9450
ARIMA-ANN	249.1046	344.5775	0.9882	0.9469
CEEMDAN-LSTM	127.0750	168.3214	0.5023	0.9879
EMD-ARMA-LSTM	181.7516	235.1773	0.7187	0.9751
CAL	120.8184	159.8226	0.4789	0.9885

**Table 6:** Prediction results of different models in S&P500.

Model	MAE	RMSE	MAPE (%)	R${}^{2}$
LSTM	33.4958	53.4345	1.1207	0.9595
ARIMA	34.1031	54.8336	1.1411	0.9598
GRU	43.3137	63.2251	1.4416	0.9469
Bi-LSTM	33.5198	53.4177	1.1262	0.9610
ARIMA-ANN	33.7170	53.6489	1.125	0.9608
CEEMDAN-LSTM	21.1496	30.1187	0.6964	0.9878
EMD-ARMA-LSTM	22.1886	33.4485	0.7334	0.9843
CAL	17.1362	26.1373	0.5645	0.9910

**Table 7:** Prediction results of different models in SSE.

Model	MAE	RMSE	MAPE (%)	R${}^{2}$
LSTM	38.3486	47.9563	1.2468	0.9475
ARIMA	25.1019	36.9815	0.819	0.9690
GRU	31.8217	43.1568	1.0355	0.9599
Bi-LSTM	31.8026	42.7439	1.0382	0.9596
ARIMA-ANN	25.6976	37.4014	0.8383	0.9686
CEEMDAN-LSTM	14.3562	19.6741	0.4681	0.9913
EMD-ARMA-LSTM	19.5074	28.5532	0.6382	0.9814
CAL	14.0294	19.9246	0.459	0.9911

**Table 8:** The regression parameters and diagnostics results.

Model	Parameter	Estimation	SE	*t*	*p*
DAX	*a*	0.9909	0.005	196.519	0.000
	*b*	104.2845	62.836	1.660	0.098
HSI	*a*	1.0012	0.006	167.616	0.000
	*b*	−12.2083	153.286	−0.080	0.937
S&P500	*a*	0.9844	0.005	192.819	0.000
	*b*	44.7296	16.195	2.762	0.006
SSE	*a*	0.9913	0.005	187.342	0.000
	*b*	28.5226	16.263	1.754	0.080

## Data Availability

Publicly available datasets were analyzed in this study. DAX data can be found here: https://cn.investing.com/indices/germany-30-historical-data. HSI data can be found here: https://cn.investing.com/indices/hang-sen-40-historical-data. S&P500 data can be found here: https://cn.investing.com/indices/us-spx-500-historical-data. SSE data can be found here: https://cn.investing.com/indices/shanghai-composite-historical-data.
